# Neuroprotection in an Experimental Model of Multiple Sclerosis via Opening of Big Conductance, Calcium-Activated Potassium Channels

**DOI:** 10.3390/ph16070972

**Published:** 2023-07-07

**Authors:** Gareth Pryce, Sofia Sisay, Gavin Giovannoni, David L. Selwood, David Baker

**Affiliations:** 1 BartsMS, The Blizard Institute, Faculty of Medicine and Dentistry, Queen Mary University of London, London E1 2AT, UK; gpryce58@gmail.com (G.P.);; 2 Wolfson Institute for Biomedical Research, University College London, London WC1E 6BT, UK

**Keywords:** autoimmunity, BK channel, experimental autoimmune encephalomyelitis, multiple sclerosis, neuroprotection

## Abstract

Big conductance calcium-activated (BK) channel openers can inhibit pathologically driven neural hyperactivity to control symptoms via hyperpolarizing signals to limit neural excitability. We hypothesized that BK channel openers would be neuroprotective during neuroinflammatory, autoimmune disease. The neurodegenerative disease was induced in a mouse experimental autoimmune encephalomyelitis model with translational value to detect neuroprotection in multiple sclerosis. Following the treatment with the BK channel openers, BMS-204253 and VSN16R, neuroprotection was assessed using subjective and objective clinical outcomes and by quantitating spinal nerve content. Treatment with BMS-204253 and VSN16R did not inhibit the development of relapsing autoimmunity, consistent with minimal channel expression via immune cells, nor did it change leukocyte levels in rodents or humans. However, it inhibited the accumulation of nerve loss and disability as a consequence of autoimmunity. Therefore, in addition to symptom control, BK channel openers have the potential to save nerves from excitotoxic damage and could be useful as either stand-alone neuroprotective agents or as add-ons to current disease-modifying treatments that block relapsing MS but do not have any direct neuroprotective activity.

## 1. Introduction

Multiple sclerosis (MS) is the major cause of non-traumatic disability in young adults. Disease induces disabling attacks and nerve damage leading to impaired neurotransmission and the development of several poorly controlled, troublesome symptoms [1]. These develop due to demyelination and nerve loss that occurs due to relapsing immune-mediated attacks and progressive MS [1,2]. To date, the control of neurodegeneration, independent of the blockade of relapses, has proved elusive. However, the blockade of excitatory ion channels has been found to inhibit neurodegeneration due to the inflammatory penumbra that occurs during relapsing neuroimmunological disease [3,4,5,6]. Compounds that limit excitatory, sodium, and calcium ion channel activity or boost inhibitory neuronal activity can be associated with sedative side effects, as they block neuronal signaling [6,7,8,9]. This can limit drug compliance and efficacy [9,10].

We have recently demonstrated that big-conductance, calcium-activated, potassium channel (BK channel, KCa1.1, gene KCNMA1) openers are well tolerated in animal models of progressive MS and humans [11]. These channels become active in high calcium ion concentrations or during marked depolarization conditions and induce membrane hyperpolarization that can limit excessive neural activity that causes spasticity and other neurological signs [11,12,13,14]. The proximity of Ca^2+^ channels to BK channels allows BK channels to sense the Ca^2+^ increase and to counteract depolarization, limiting pathological neurodegenerative activity [12,13]. BK channels directly interact with myelin basic protein (MBP), and MBP increases the Ca^2+^ sensitivity [15]. The BK channel contributes to cell excitability in unmyelinated axons [16]. Furthermore, BK channels may limit excitotoxicity, which can cause neurodegeneration in various conditions, including MS and epilepsy [13,17,18]. Indeed, it has been shown that BMS-204352, a BK channel isoform, non-selective (KCNMA1) opener [19,20], can be neuroprotective in several different experimental assays, including brain trauma, spinal cord injury, and ischemia [20,21,22].

Whilst BK channel subunits are expressed by several cell types [14,23], there is a relative absence of BK channels in immune cells [24]. In neuronal tissues, the predominant subunit combinations are α1β2 and α1β4 [25]. It was hypothesized that R, Z)-3-(6-(dimethylamino)-6-oxohex-1-enyl)-N-(1-hydroxypropan-2-yl) benzamide (VSN16R), a novel orally active, neuronally selective (no activity against the smooth muscle forms α1β1) BK channel opener [11,26], may therefore also exhibit neuroprotective potential in MS, in the absence of immunosuppressive activity. This was assessed in an experimental autoimmune encephalomyelitis (EAE) model of MS [27], which has shown translational value in detecting neuroprotective agents in multiple sclerosis [5,6].

## 2. Results

### 2.1. Tissue Expression of Neural BK Channel

**Figure 1:**
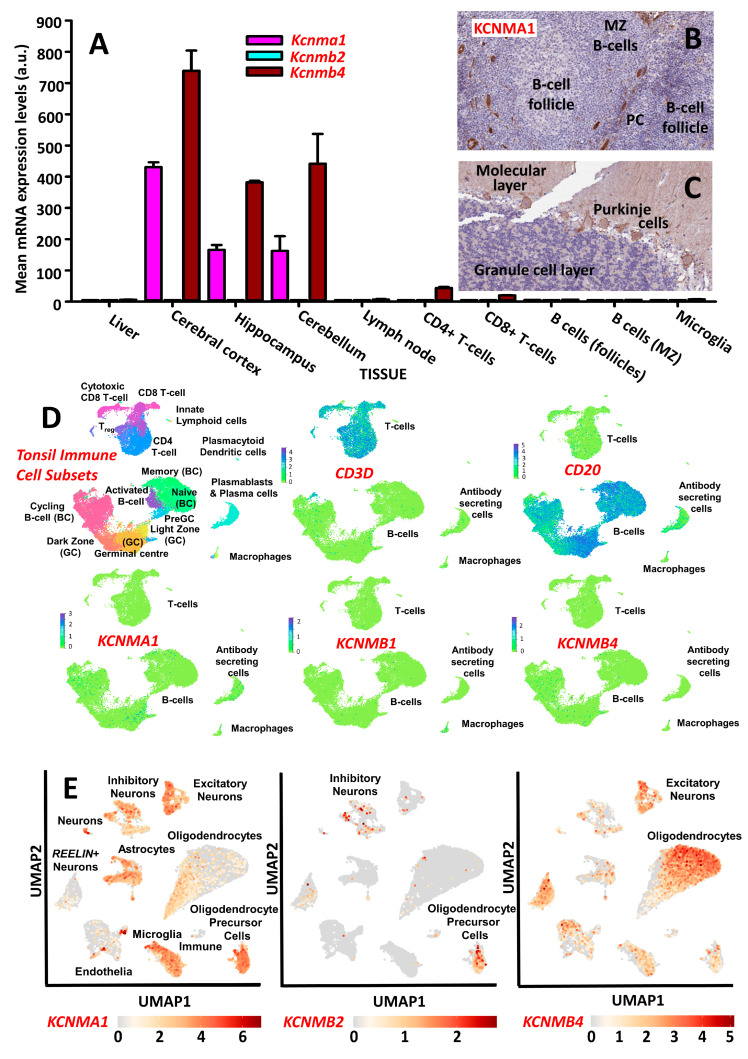
Lymphocytes exhibit limited BK channel expression. Tissue expression of BK channel subunits was extracted from public databases under Creative Commons licenses (**A**) Messenger RNA expression of mouse *Kcnma1* (1424848_at), *Kcnmb2* (1431844_at) and *Kcnmb4* (144941_at) data were extracted from the mouse Gene Atlas MOE430, gcrma following RNAseq using Affymetrix MOE430_2 microarrays (www.biogps.org (accessed on 6 July 2023) [28,29,30]). The results represent mean ± SD expression in arbitrary units (a.u.). (**B**,**C**) Data were extracted from the Human Protein atlas [32,33] KCNMA1 protein expression was assessed using immunoperoxidase staining of sections in (**B**) human lymph node (https://www.proteinatlas.org/ENSG00000156113-KCNMA1/tissue/lymph+node#img (accessed on 6 July 2023)) and (**C**) human cerebellum (https://www.proteinatlas.org/ENSG00000156113-KCNMA1/tissue/cerebellum#img (accessed on 6 July 2023)). Follicular and marginal zone (MZ) B-cells and paracortical (PC) T-cells exhibited no or low-level expression of *KCNMA1*. Expression was detected in lymph node blood vessels consistent with vascular *KCNMA1* and *KCNMB1* expression and was evident in the grey matter within the cerebellum. (**D**) Single-cell RNAseq expression data of BK channel subunits within human tonsils were extracted from public databases and assessed using Illumina NextSeq 50010X. The results represent Uniform manifold approximation and projection (UMAP) plots from data based on results from up to 32,000 cells from 7 donors, showing the distribution in immune subsets and expression of *CD3D* (T cell marker), *CD20/MSA41* (B cell marker), and *KCNMA1*, *KCNMB1*, and *KCNMB4*. (www/tonsilimmune.org (accessed on 6 July 2023) [34]). (**E**) Single-cell RNAseq expression data of BK channel subunits within human brain was extracted from public databases [35] and assessed using 10X Genomics Chromium single cell 3′ chips. Figures show UMAP representations of *KCNMA1*, *KNCMB2*, and *KCNMB4* in different cell lineages.

**Figure 2:**
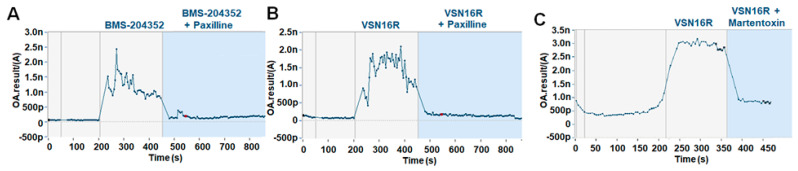
Inhibition of VSN16R potassium channel opening using BK channel antagonists. Automated whole-cell analysis of individual human Ea.hy926 cells demonstrating the influence of 15 μM (**A**) BMS-204352 or (**B**) VSN16R alone or with co-administration with 2 μM paxilline or (**C**) 100 nM martentoxin.

BK channel gene expression from public databases was analyzed [28,29,30,31,32,33,34]. The analysis indicated that mouse leukocytes, including splenocytes, CD4^+^ T cells, CD8^+^ T cells, and B220 (CD45RA)^+^ B cells, and macrophages expressed minimal *Kcnma1* and *Kcnmb4* compared to brain tissue (Figure 1A) [28,29,30]. This was consistent with the minimal expression of KCNMB4 message in human leukocytes in the barcode of normal tissues and Gene Atlas U133A data sets using microarray (probe 219287_at. www.biogps.org (accessed on 6 July 2023)) [28,30,31]. This expression confirmed previous polymerase chain reaction analysis, which indicated that spleens exhibited negligible *Kcnma1* expression compared to brain tissue [24]. This was consistent with the immunocytochemistry of human lymph nodes [32,33], where the expression of KCNMA1 is limited to the vasculature and not the T or B cells within the cortex, follicles, and paracortex (Figure 1B,C). The relative lack of immune cells was also evident following the extraction of single-cell sequence data from human tonsil tissue (Figure 1D) [34]. KCNMB4 was detected on oligodendrocytes using single cell seq (Figure 1E) [23,35]. It is also expressed via neurons [24,28,30,31], where it was notably found on glutamatergic, excitatory nerves, compared to KCNMB2, which was more common on GABAergic, inhibitory nerves (Figure 1E) [14,35]. This is consistent with the ability of KCNMB4 openers to act as a symptomatic treatment [11,14]. This suggests the potential to limit excitotoxicity without affecting other systems. Consistent with this, BMS-204352 [19] and VSN16R [11] are BK channel openers that are inhibited via the action of paxilline (Figure 2A,B), which can augment potassium-mediated hyperpolarizing currents [11]. In addition, martentoxin, a KCNMA1^+^, KCNMB4^+^ channel antagonist [36], was found to inhibit responses (Figure 2C). However, this was not universal on all cells suggesting a complex, perhaps state-dependent, effect on the KCNMB4-expressing cells [11] used in the assay. The relative lack of KCNMA1/Kcnma1 on leukocytes suggested that VSN16R would not be immunosuppressive.

### 2.2. VSN16R Does Not Induce Immunosuppression

Although doses ≥1 mg/kg VSN16R p.o. can inhibit spasticity in EAE in ABH mice, mice can tolerate over 1000 mg/kg p.o. in the absence of any sedative effects [11]. Likewise, VSN16R was well tolerated in Wistar rats and Beagle dogs [11]. Consistent with the limited expression of any BK channel isoform via the immune system (Figure 1A–D), daily oral doses of up to 1000 mg/kg/day in rats and 200 mg/kg/day in dogs, which were the maximum doses tested, did not induce any marked effect on leukocyte subsets detected in the blood of rats (Table 1A) or dogs (Table 1B) when treated for 28 days. Likewise, there was no influence of leukocyte subsets in healthy human volunteers (Table 1C) when treated with 800 mg VSN16R (10 mg/kg) in gelatine capsules following a single dose in humans [11]. This also was evident when humans were treated twice daily with 400 mg VSN16R, which produced plasma levels far exceeding that induced with comparable doses in mice [11] for one week (Table 1C).

**Figure 3:**
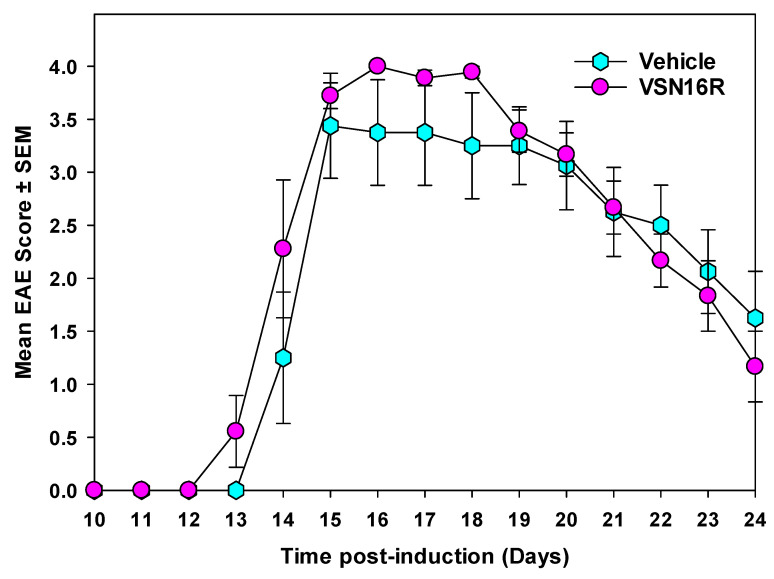
VSN16R is not immunosuppressive during the initial attack of EAE. Adult ABH mice were injected with spinal cord homogenate in Freund’s adjuvant on days 0 and 7. Animals were treated daily with either vehicle (0.1 mL water. Hexagon *n* = 8) or 40 mg/kg p.o. VSN16R in water (circle *n* = 10) from day 10 onwards. Clinical signs were grade 0 = normal, 1 = limb tail, 2 = impaired righting reflex, 3 = hind limb paresis, 4 = hind limb paralysis, and 5 = moribund. The results represent the mean ± SEM daily score of all animals developing neurological disease.

Following experimental autoimmune encephalomyelitis (EAE) induction in Biozzi ABH mice via sensitization with mouse spinal cord homogenate in Freund’s adjuvant [27], it was found that the daily treatment with 40 mg/kg p.o. VSN16R does not (*p* > 0.05) influence the development of EAE (Table 2, Figure 3). As such, VSN16R did not reduce the incidence, severity, or onset of EAE (Table 2) in a treatment paradigm where potent immunosuppressive agents can completely inhibit the development of EAE and all its downstream consequences [37,38]. Therefore, the neuroprotective effect of VSN16R against the inflammatory penumbra that develops during relapsing disease could be analyzed in an induced-relapse paradigm [5,39].

### 2.3. VSN16R and Neuroprotection

**Figure 4:**
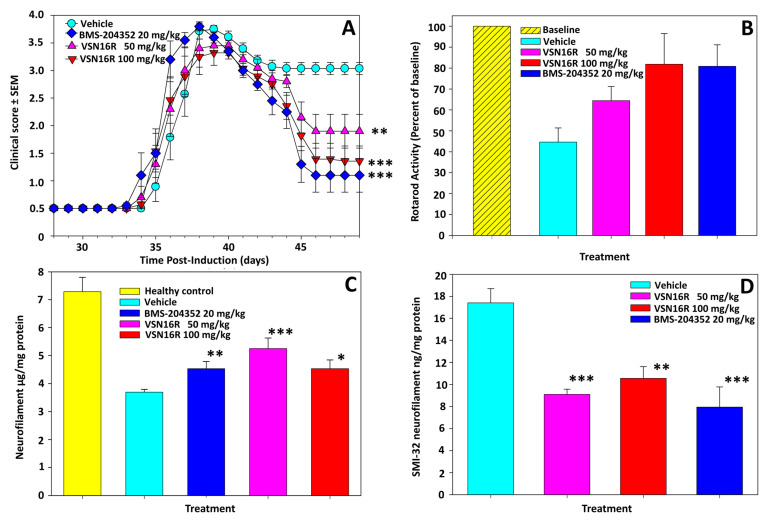
VSN16R is not immunosuppressive in the relapse phase of EAE but is neuroprotective. Adult ABH mice were injected with spinal cord homogenate in Freund’s adjuvant on days 0 and 7 and on day 28 to induce a relapse. Following accelerating-rotarod analysis on day 27, animals were randomized to either 0.1 mL water (circle), 50 mg/kg p.o. VSN16R (triangle), 100 mg/kg p.o. VSN16R in water (inverse triangle), or 20 mg/kg i.p. BMS-204352 (diamond) in DMSO: cremophor: PBS (1:1:18. N = 15/group). Rotarod activity was retested on day 48 post-inoculation. The results represent (**A**) The mean ± SEM daily score of animals developing neurological disease graded: 0 = normal, 1 = limb tail, 2 = impaired righting reflex, 3 = hind limb paresis, 4 = hind limb paralysis, 5 = moribund (**B**) The mean ± SEM percentage loss of motor co-ordination due to relapse, based on time to fall from an accelerating rotarod, compared to baseline on day 28. (**C**) The mean ± SEM total neurofilament level within the whole spinal was assessed using a neurofilament-specific ELISA. (**D**) The mean ± SEM total, hypo-phosphorylated neurofilament levels within the whole spinal were assessed using an SMI32-specific ELISA. * *p* < 0.05, ** *p* < 0.01, *** *p* < 0.001 compared to vehicle control.

Although VSN16R can enter the central nervous system (CNS) and notably targets active lesions [11], BMS-204352 is hydrophobic and readily enters the CNS [20,40]. BMS-204352 likewise failed to inhibit the development of relapsing disease, further indicating that BK channel openers are not immunosuppressive (Table 2). To assess the neuroprotective potential of BK channel openers, damaging relapses were induced via an additional boost with an antigen (Figure 4. Table 2). Both 50 mg/kg and 100 mg/kg p.o. VSN16R and 20 mg/kg i.p. BMS-204352 did not inhibit the development of relapse (Figure 4A. Table 2), as assessed by the maximum severity of the disease and disease onset. However, they notably limited the accumulation of neurological deficit as a consequence of the attack and exhibited a significantly (*p* < 0.01-*p* < 0.001) better clinical recovery compared to vehicle-treated animals (Figure 4A). This was also evident with the less deficit accumulated on accelerating-rotarod activity (Figure 4B) and spinal nerve content as assessed using a quantitative, neurofilament-specific ELISA (Figure 4C). Furthermore, of the surviving nerves, the BK channel opening protected against the development of dystrophic nerves indicated but reduced production of non-phosphorylated neurofilament (Figure 4D). This indicates that BK channel channels are neuroprotective against the inflammatory penumbra, where active inflammation induces neurological damage. There was a suggested dose response with VSN16R, and 100 mg/kg VSN16R exhibited similar neuroprotective activity to 20 mg/kg BMS-204352 (Figure 4).

## 3. Discussion

This study demonstrates that BK potassium channel openers have neuroprotective potential in neuroinflammatory disease and could be a novel, non-sedating method to target pathological neural hyper-excitability and excitotoxicity in MS. The lack of immunosuppressive activity is consistent with the relative lack of expression of the BK channel via lymphocytes [24] and contrasts with the reported immunosuppressive effects of blockers of a number of different potassium channels [41,42,43]. The neuroprotective effect, independent of immunosuppression, is consistent with the neuroprotection observed following brain trauma, spinal cord injury, and ischemia [20,21,22,44]. This benefit may occur via activity on multiple complementary pathways, including the blockage of excitotoxicity and the promotion of anti-oxidant properties [20,21,22,44,45].

Sodium channel blockade has been suggested to act via inhibition of microglial function in addition to effects on nerves [3,4]. Microglia appear to have a more limited expression of KCNMA1-KCNMB4 than those found in nerves [46]. However, it has been shown that BK channel openers directly block glutamate excitotoxicity in nerves, via the opening of the KCNMA1-formed channel, possibly via activity on the mitochondrial BK channel channels [47]. It is perhaps of interest that oligodendrocytes and notably oligodendrocyte precursor cells, which exhibit ion-channel-related myelinating activities [48] express the neuronal KCNMA1, KCNMB4 isoform [23,46]. These cells are also sensitive to potassium-loading, induced death, and oligodendrocyte excitotoxicity [49,50]. Direct physical contact between MBP and KCNMA1 has been noted, and MBP increases the Ca^2+^ sensitivity of the BK channel [15]. As anandamide, which activates the BK channel, can protect oligodendrocytes [50,51], it is possible that VSN16R and BMS-204352 may also limit oligodendrocyte damage, and this requires further study [23].

Most excitatory ion channel modulators, including the blockers of glutamate receptors, have sedative or other undesirable psychotropic effects [8,9,52]. However, as the BK channels are only opened once, high intracellular concentrations of calcium are detected via intracellular Ca^2+^ sensors or marked membrane depolarization [12,13]. This suggests that the drug target may only appear during potentially pathological conditions. This may contribute to the finding that BK channel openers are well tolerated in animals and humans and do not tend to inhibit normal behaviors [11,14,19]. That deficits in BK channel channels have been associated with seizures and conditions associated with nerve over-excitability [13,18,40] further supports the view that BK channel control neural excitability and consequent excitotoxicity, consistent with the cellular distribution of glutamatergic and GABAergic neurons [14].

Although it will take considerable time to develop neuroprotective agents in MS, as phase III neuroprotective studies take years to recruit and undertake [53], a more direct route to the proof of concept exists via trialing in optic neuritis and monitoring with optical coherence tomography [54]. BK channel openers could more rapidly become available to treat multiple sclerosis-related symptoms [11,55]. The lack of immunosuppressive activity means that such agents could be useful add-ons to current disease-modifying treatments, which block peripheral immunity but offer limited direct neuroprotective activity.

## 4. Materials and Methods

### 4.1. Chemicals

VSN16R [11,56] was synthesized by Park Place Research (Cardiff, UK) or Dalton Chemical Laboratories Inc. (Toronto, ON, Canada). BMS-204352 (Maxipost. (3S)-(+)-(5-Chloro-2-methoxyphenyl)-1,3-dihydro-3-fluoro-6-(trifluoromethyl)-2H-indole-2-one [19] and the BK channel antagonist Paxilline were purchased from Tocris Ltd. (Bristol, UK). Martentoxin, the KCNMBA1,KCNMB4-selective scorpion toxin [36], was synthesized according to the FGLIDVKCFASSECWTACKKVTGSGQGKCQNNQCRCY sequence [57] by Peptide-Protein Research Ltd., Fareham, UK. This sequence contains the N-terminal phenylalanine, which may be lacking from some commercial suppliers but is critical for Martentoxin function [24].

### 4.2. Humans

A double-blind, placebo-controlled phase I safety study was performed on healthy volunteers (EudraCT 2013-002765-18) following ethical review (CPMP/ICH/135/95) and informed consent that complied with the Declaration of Helsinki. Individuals (*n* = 6) received twice daily 25 mg, 100 mg, and 400 mg VSN16R (maximum dose ~10 mg/kg/day; maximum dose tested) as part of a multiple ascending dose, phase I safety study in healthy humans [11]. Serial plasma samples using EDTA anti-coagulant were collected. Quintiles Limited, London, UK, performed this study. The sample size was based on experience from previous similar phase I studies [11].

### 4.3. Animals

Male and female Biozzi ABH mice were bred and used at Queen Mary University of London under the European Union Directives 2010/63/EU and Animals (Scientific Procedures) Act 1986. Protocols relating to the ARRIVE guidelines have been reported previously [27]. Rat and dog studies were performed as part of pre-clinical toxicology testing by Charles Rivers UK [11].

### 4.4. Tissue Expression

BK channel gene expression was extracted from data in public databases: BioGPS www.biogps.org [28,29,30,31]; Human protein atlas. www.proteinatlas.org [32,33], Immune cell atlas of the human tonsil. www.tonsilimmune.org (accessed on 6 July 2023) [34] and the Human white matter cell heterogeneity with region, age, and sex atlas. https://seeker-science.shinyapps.io/shiny_app_multi/ (accessed on 6 July 2023)) [35] under the Creative Commons Attribution-Share Alike 3.0 International License, (https://creativecommons.org/licenses/by-sa/3.0/ (accessed on 6 July 2023)), the Creative Commons Attribution 4.0 International License (http://creativecommons.org/licenses/by/4.0/ (accessed on 4 July 2023)), and the Creative Commons Public Domain Dedication waiver (http://creativecommons.org/publicdomain/zero/1.0/ (accessed on 6 July 2023)).

### 4.5. Electrophysiology

A stepped, whole-cell recording electrophysiology assay for BK channel activity was generated in BK channel expressing human Ea.hy926 cell lines (American Type Tissue Culture CRL-2922, Lot:63777495. Baker et al., 2017 [11]) seeded in SyncroPatch 384PE plates (Nanion Technologies, Munich, Germany) and assayed in wells achieving a seal resistance of >500 MΩ and validated using activators, BMS-204532 (15 µM) and the inhibitor Paxilline (2 µM). Whole-cell recordings of BK channel channels used a pipette solution containing 145 mM KCl, 10 mM HEPES, 1 mM MgCl_2_, and 5 mM EGTA, and free Ca^2+^ concentration was set to 300 nM by adding 3.27 mM CaCl_2_. The bath K^+^-based solutions with 300 nM free Ca^2+^ were used for recordings.

### 4.6. Induction of Experimental Autoimmune Encephalomyelitis

Adult ABH mice were injected under the skin of the flank with 1 mg mouse spinal cord homogenate (SCH) emulsified in Freund’s complete adjuvant containing 60 μg *Mycobacterium tuberculosis* H37Ra and *M. butyricum* (8:1) in the flank on day 0 and 7, as described previously [27]. The disease was scored: Normal = 0; Fully flaccid tail = 1; Impaired righting reflex = 2; Hind limb paresis = 3; Complete hind limb paralysis = 4, and Moribund/death = 5 [27]. The initial disease episode occurred around day 15–20 post-induction. To induce neurodegeneration associated with the inflammatory penumbra of an attack, a relapse was induced on day 28 post-induction following an additional injection of SCH in Freund’s adjuvant [5,27]. Rotarod-assessed motor-co-ordination was measured using an accelerating (4–40 revolutions per minute) rotarod (Med Associates, Fairfax, VT, USA) on day 27 and day 48 post-induction [27]. Details of randomization, blinding and sample size calculations, and other experimental details relevant to the ARRIVE guidelines have been reported previously [27]. In the neuroprotection studies, animals were allocated to groups on day 27, based on rotarod activity (highest to lowest sequentially for each group) and assessed blinded to treatment prior to daily drug delivery. Spinal cord nerve content was assessed using a quantitative neurofilament-specific ELISA, using bovine neurofilament as standard, on whole spinal cords expelled from the spinal column using hydrostatic pressure [27,39]. This offers advantages over point-in-time histology, as it is a clinically translatable, quantitative outcome that measures the accumulated influences occurring throughout the spinal cord in an unbiased way [10,58]. The presence of dystrophic nerves was assessed using a hypo-phosphorylated neurofilament (SMI-32)-specific ELISA, as described previously [39]. Using SCH as an immunogen precludes ex vivo analysis as SCH-sensitized animals fail to give robust T cell responses to the pathologically dominant myelin epitopes.

### 4.7. Drug Treatment

VSN16R doses in Wistar rats and Beagle dogs represent the highest No Adverse Event Level tested in toxicology studies undertaken by Charles Rivers, UK [11]. For mouse studies, VSN16R was dissolved in distilled water and sonicated for 10 min using a Bransonic ultrasonic cleaner prior to oral delivery of 0.1 mL. A relatively high oral dose (50 mg/kg or 100 mg/kg) of VSN16R was selected due to the: high drug-tolerability, partial CNS, relatively-short elimination half-life [11], and the aim to deliver once a day. BMS-204352 was dissolved in dimethyl sulphoxide (Sigma-Aldrich, Poole, UK) DMSO, then cremophor (Sigma) and PBS (1:1:18) and delivered via intraperitoneal injection. The dose was selected to be a high dose, below the 60 mg/kg i.p., causing sedation [59].

### 4.8. Statistical Analysis

Results are expressed as mean ± SEM unless otherwise stated, and differences between clinical scores were assessed via Mann–Whitney U statistics using SigmaPlot software (Systat Software, Hounslow, UK). Differences between rotarod and neurofilament levels were assessed using *t*-tests following the assessment of the normal distribution of data and equality of variances using Sigmaplot.

## 5. Conclusions

This study indicates that BK potassium channel openers are well tolerated agents that exhibit neuroprotection without inducing marked immunosuppression during neuroinflammatory disease. Therefore, this class of agents may be a useful, novel addition to current therapies for the treatment of multiple sclerosis.

## Figures and Tables

**Table 1:** VSN16R does not deplete leukocytes in rats, dogs, and humans.

Treatment	Sex	*n*	RBC ×10^−12^/µL	Cell Numbers × 10^−9^/µL						
				WBC	Neutrophils	Lymphocytes	Monocytes	Eosinophils	Basophils	Platelets
A. Rats Treated for 4 Weeks QD										
Vehicle 100 mg/kg 300 mg/kg 1000 mg/kg	Male Female Male Female Male Female Male Female	10 9 8 10 9 9 8 10	7.86 ± 0.23 7.76 ± 0.20 7.78 ± 0.28 7.77 ± 0.34 7.84 ± 0.34 7.61 ± 0.27 7.97 ± 0.24 7.70 ± 0.16	12.19 ± 2.64 8.79 ± 2.67 10.28 ± 1.40 7.47 ± 1.58 13.12 ± 2.41 9.92 ± 4.09 12.63 ± 2.89 9.09 ± 2.55	1.52 ± 0.79 0.67 ± 0.18 1.10 ± 0.32 0.91 ± 0.52 2.03 ± 1.71 0.86 ± 0.29 1.70 ± 0.83 0.73 ± 0.31	9.99 ± 2.15 7.74 ± 2.54 8.47 ± 1.52 6.21 ± 1.11 10.33 ± 0.98 8.64 ± 3.80 10.24 ± 2.36 8.02 ± 2.30	0.24 ± 0.07 0.16 ± 0.04 0.24 ± 0.07 0.12 ± 0.05 0.29 ± 0.17 0.14 ± 0.11 0.27 ± 0.11 0.13 ± 0.08	0.12 ± 0.04 0.15 ± 0.05 0.16 ± 0.06 0.17 ± 0.08 0.12 ± 0.06 0.18 ± 0.12 0.12 ± 0.04 0.12 ± 0.0	0.16 ± 0.04 0.15 ± 0.05 0.17 ± 0.04 0.17 ± 0.08 0.19 ± 0.06 0.18 ± 0.12 0.18 ± 0.07 0.12 ± 0.06	894 ± 148 969 ± 162 1041 ± 184 892 ± 136 956 ± 275 1202 ± 526 1103 ± 181 1112 ± 190
B. Dogs Treated for 4 Weeks QD										
Vehicle 50 mg/kg 100 mg/kg 200 mg/kg	Male Female Male Female Male Female Male Female	3 3 3 3 3 3 3 3	7.17 ± 0.28 6.75 ± 0.61 7.07 ± 0.45 6.90 ± 0.48 6.89 ± 0.24 7.04 ± 0.20 7.20 ± 0.30 6.47 ± 0.48	9.89 ± 1.34 9.15 ± 1.40 9.60 ± 2.58 10.87 ± 1.88 9.78 ± 1.55 9.58 ± 1.85 9.61 ± 0.58 9.34 ± 1.39	6.60 ± 1.15 5.37 ± 0.68 6.19 ± 1.97 7.24 ± 1.13 5.89 ± 1.00 6.23 ± 1.31 5.87 ± 1.97 6.00 ± 1.08	2.38 ± 0.32 2.89 ± 0.71 2.46 ± 0.43 2.53 ± 0.79 2.76 ± 0.46 2.51 ± 0.45 2.68 ± 0.51 2.47 ± 0.24	0.52 ± 0.13 0.45 ± 0.06 0.53 ± 0.18 0.44 ± 0.08 0.61 ± 0.05 0.45 ± 0.13 0.51 ± 0.06 0.49 ± 0.14	0.17 ± 0.14 0.21 ± 0.07 0.21 ± 0.09 0.29 ± 0.15 0.26 ± 0.13 0.14 ± 0.08 0.23 ± 0.12 0.19 ± 0.05	0.19 ± 0.04 0.20 ± 0.09 0.18 ± 0.04 0.32 ± 0.01 * 0.23 ± 0.06 0.22 ± 0.07 0.27 ± 0.06 0.16 ± 0.04	333 ± 37 337 ± 37 369 ± 41 458 ± 87 299 ± 124 397 ± 49 341 ± 20 358 ± 47
C. Humans Treated for 1 Week BID										
Placebo ~0.8 mg/kg ~3.3 mg/kg ~13 mg/kg	Male Male Male Male	6 6 6 6	4.78 ± 0.32 5.00 ± 0.28 4.76 ± 0.22 4.78 ± 0.32	6.70 ± 1.40 5.00 ± 1.00 5.90 ± 1.70 6.70 ± 1.40	3.40 ± 1.20 2.10 ± 0.50 3.30 ± 1.40 3.40 ± 1.20	2.50 ± 0.70 2.00 ± 0.50 1.90 ± 0.60 2.50 ± 0.70	0.60 ± 0.10 0.60 ± 0.10 0.50 ± 0.20 0.60 ± 0.10	0.20 ± 0.10 0.20 ± 0.20 0.20 ± 0.20 0.20 ± 0.10	0.00 ± 0.10 0.00 ± 0.10 0.00 ± 0.00 0.00 ± 0.10	254 ± 47 220 ± 35 204 ± 49 254 ± 47

(A) Sprague Dawley rats and (B) Beagle dogs were administered orally various amounts of VSN16R in water for 28 days following collection of blood, red blood cells (RBC), white blood cells (WBC), and leukocyte subset cell numbers were assessed. (C) Healthy humans received VSN16R in gelatine capsules or placebo as part of phase I safety studies at 25 mg, 100 mg, and 400 mg twice daily. * *p* < 0.05.

**Table 2:** BK channel openers are not immunosuppressive in relapsing EAE.

Treatment	Dose	No. EAE/Total	Group Score	EAE Score	Day of Onset
Initial Paralytic Episode					
Untreated Vehicle VSN16R	- 0.1 mL H_2_O p.o. 40 mg/kg p.o.	12/12 8/9 10/10	3.7 ± 0.2 3.5 ± 0.4 4.0 ± 0.0	3.7 ± 0.2 3.9 ± 0.1 4.0 ± 0.0	14.6 ± 2.8 15.0 ± 1.7 13.6 ± 1.5
Induced-Relapse					
Vehicle BMS-204352	0.1 mL DCP i.p. 20 mg/kg i.p.	12/12 10/10	4.0 ± 0.0 4.0 ± 0.0	4.0 ± 0.0 4.0 ± 0.0	36.3 ± 1.2 36.3 ± 0.9
Vehicle VSN16R VSN16R BMS-204352	0.1 mL H_2_O p.o. 50 mg/kg p.o. 100 mg/kg p.o. 20 mg/kg i.p.	16/16 15/15 14/15 14/15	3.9 ± 0.1 3.9 ± 0.2 3.5 ± 0.2 3.7 ± 0.0	3.9 ± 0.1 3.9 ± 0.2 3.8 ± 0.1 3.9 ± 0.0	36.9 ± 1.3 36.4 ± 1.5 36.1 ± 2.1 35.5 ± 1.1

Adult ABH mice were injected with spinal cord homogenate in Freund’s complete adjuvant on days 0 and 7. Animals were treated daily with either vehicle (water) or 40 mg/kg p.o. VSN16R in water (circle) from day 10 onwards. Following recovery from the initial attack, animals remitted by about day 21–24, and a relapse was induced via a further injection of spinal cord autoantigens in Freund’s incomplete adjuvant, and treatment was initiated on day 34 post-inoculation, 1–2 days before the anticipated relapse. Animals were injected daily with BMS-204352 or DMSO, cremophor phosphate-buffered saline (1:1:18. DCP), or were administered VSN16R or water vehicle orally. Blocks of data represent individual experiments. Clinical signs were grade 0 = normal, 1 = limb tail, 2 = impaired righting reflex, 3 = hind limb paresis, 4 = hind limb paralysis, and 5 = moribund. The results represent the mean maximum, individual, clinical score ± SEM of all animals within the Group (Group Score) or only those developing clinical EAE, excluding the non-susceptible animals (EAE Score) and the mean ± SD day of onset of clinical signs.

## Data Availability

Data is available on reasonable request to senior authors.
